# The Relationship between Peritoneum and Body Organs in Persian Medicine

**Published:** 2018-07

**Authors:** Bagher MINAEI, Farshad AMINI-BEHBAHANI, Mohsen BAHRAMI, Behzad EFTEKHAR, Abdolghader BAHRAINI, Majid DADMEHR

**Affiliations:** 1. Dept. of Histology, School of Medicine, Tehran University of Medical Sciences, Tehran, Iran; 2. Research Institute for Islamic and Complementary Medicine, Iran University of Medical Sciences, Tehran, Iran; 3. School of Persian Medicine, Iran University of Medical Sciences, Tehran, Iran; 4. Dept. of Neurosurgery, Australian School of Advanced Medicine, Macquarie University, Sydney, Australia; 5. Dept. of Neurosurgery, Nepean Hospital, University of Sydney, New South Wales, Australia; 6. Student Research Committee, Iran University of Medical Sciences, Tehran, Iran

## Dear Editor-in-Chief

Fascia is the soft tissue component of the connective tissue system that encloses and connects muscles, blood vessels, nerves and all structures of the body; it extends from head to toe and creates a continuous network throughout the body. Fascia is a dynamic and active structure which contains a rich vascular and nerve supply and intimately connects with the autonomic nervous system. It plays a mediating role which able to transmit the electrical signals and mechanical tensions in human body. Moreover, fascia is closely related to nourishment of all cells in the body, so any functional impairment in the fascia can have serious effects on human health (1–3).

Physicians of ancient civilization recognized fascia and it is a widely used anatomical term. In the Persian medicine (PM), anatomy and functions of the fascia are well considered. PM scholars have defined a membranous structure in the abdomen called “*Maraqq*” (4–6). It is characterized as an external [parietal] layer of peritoneum (4,6) and a fascia surrounding of digestive tract (stomach, liver, spleen, intestine, etc.) (5,6). In some other texts, this term refers only to the abdominal skin (6), moreover, external layer of peritoneum with muscles and skin together (4) and all of the abdominal wall layers are considered as the “*Maraqq*” (7).

According to PM “*Maraqq*” is classified as a fascia (4–6) which is a nerve-like (white, firm and flexible), thin and translucent tissue and encloses the outer surface of the body organs (4).

Moreover, “*Maraqq*” has a relationship with other membranes of the body such as chest fascia; it is mentioned as an origin of the chest fascia (4). This membrane naturally interferes with the physiological functions of the body; some of them include contribution to peristalsis, urination, exodus of bloating and uterine contractions (4).

There is an association “*Mosharekat*” among “*Maraqq*” and other body organs which participates in the pathogenesis of some diseases, PM scholars have believed that a number of brain diseases such as melancholia, epilepsy, headache, vertigo, and dizziness are related to the pathological changes in the “*Maraqq*” (4–6). In each of these diseases, the patient may experience *Maraqq*-related symptoms, in addition to the clinical manifestations of each disease which mentioned by details in ancient medical textbooks (4–6). These clinical manifestations include: sour eructation, pain, burning and distention in the stomach and the “*Maraqq*”, drooling, abdominal flatulence, mucoid stool, pain between two shoulders (4–6), heaviness in the “*Maraqq*”, borborygmus, nausea, dyspepsia (4,5) polyphagia (5,6) and slimming (6).

Furthermore, “*Maraqq*” plays a role in treatment modality of several diseases. The Persian medicine scholars are considered some treatment methods on the “*Maraqq*” for other body organs’ diseases, in addition to the specific treatments of each disease separately. Some remedies are recommended in these cases, which are including:

- Using dry cupping on the “*Maraqq*” [abdomen] in treatment of epilepsy, arterial epistaxis, abdominal pain due to flatus and uterine prolapse (5).
- Rubbing and anointing oil on the “*Maraqq*” [abdomen] in treatment of lienteric diarrhea, flatulence, constipation and bladder prolapse (4,5).
- A wrapped warm wheat bran and salt in a cloth and put it on the “*Maraqq*” [abdomen] for treatment of flatulence and constipation (5).

The relationship among “*Maraqq*” and other body organs was through the fasciae and nerve sheaths (4–7). “*Maraqq*” has a moist, loose and expandable tissue, thus, it can translocate some substances like wind “*Reeh”* and vapor *“Bokhar”* toward body organs (4). “*Reeh*” is a current flow equivalent of the wind in the nature which naturally creates in human body and plays a significant role in physiological conditions such as excretory functions in gastrointestinal and urinary tracts, any kind of quality or quantity changes of the “*Reeh*”, can disrupt the normal function of the body and disease may appear (8). In addition, “Bokhar” is caused by the effect of heat on the wet substance which could rise upward (4).

“*Maraqq*” is a fascia which plays a physiologic and mediating role in human body and can translocate special substances through the fasciae and nerve sheaths; it contributes to the pathogenesis of a number of disorders. Moreover, it has been considered on treatment plans of other body organs disorders which may be potential of introducing new treatment strategies.
